# Strong Pinned-Spin-Mediated Memory Effect in NiO Nanoparticles

**DOI:** 10.1186/s11671-017-1988-x

**Published:** 2017-03-21

**Authors:** Ashish Chhaganlal Gandhi, Ting Shan Chan, Jayashree Pant, Sheng Yun Wu

**Affiliations:** 1 Department of Physics, National Dong Hwa University, Hualien, 97401 Taiwan; 2 Center for Condensed Matter Sciences, National Taiwan University, Taipei, Taiwan; 3 National Synchrotron Radiation Research Center, Hsinchu, 30076 Taiwan; 4 Department of Physics, Abasaheb Garware College, Savitribai Phule Pune University, Pune, India

**Keywords:** NiO nanoparticles, Interfacial pinned spins, Memory effect

## Abstract

After a decade of effort, a large number of magnetic memory nanoparticles with different sizes and core/shell compositions have been developed. While the field-cooling memory effect is often attributed to particle size and distribution effects, other magnetic coupling parameters such as inter- and intra-coupling strength, exchange bias, interfacial pinned spins, and the crystallinity of the nanoparticles also have a significant influence on magnetization properties and mechanisms. In this study, we used the analysis of static- and dynamic-magnetization measurements to investigate NiO nanoparticles with different sizes and discussed how these field-cooling strengths affect their memory properties. We conclude that the observed field-cooling memory effect from bare, small size NiO nanoparticles arises because of the unidirectional anisotropy which is mediated by the interfacial strongly pinned spins.

**Electronic supplementary material:**

The online version of this article (doi:10.1186/s11671-017-1988-x) contains supplementary material, which is available to authorized users.

## Background

In recent years, NiO nanostructures have been the object of a significant amount of fundamental research because of their many uses and technological applications such as in spintronics [1], catalysis [2], anodic electrochromic materials [3], fuel/solar cells [4], supercapacitors [5], and biosensors [6]. Many of the above applications are based on the magnetic properties of the nanostructures, which vary with the synthesis method [7, 8], interparticle interaction [9], vacancies [10], and nanoparticle size [11], and show a pronounced effect on the magnetic properties of NiO nanoparticles. NiO, which is antiferromagnetic in its bulk form, exhibits ferromagnetic behavior at the nanoscale due to nickel vacancy defects, with its net magnetic moment increasing monotonically with decreasing particle size [7, 8]. Below a critical size of ~10 nm, as a consequence of the enhanced nickel vacancies, the Ni^2+^–O^2−^–Ni^2+^ superexchange interaction breaks down and the particles exhibit paramagnetic behavior at room temperature and superparamagnetic properties at lower temperature [11, 12]. The observed conventional exchange bias in Ni/NiO nanoparticles has been explained in a previous report by using a core/shell model with a higher concentration of nickel vacancies residing on the surface than in the core [7, 8]. The field-cooling hysteresis loop has also been shown to shift vertically when measured below the freezing temperature (*T*_f_) [8], which can be assigned as due to the interfacial frozen spins originating from the strong pinning effect between Ni and NiO [13]. A similar interfacial frozen-spin-mediated exchange bias effect has also been observed from Fe/Fe_3_O_4_ core/shell and Fe_3_O_4_ hollow-shell nanoparticles [14, 15]. Such pinned spins at the interface can further enhance the unidirectional anisotropy in the core/shell nanoparticles. In a recent study of the magnetic size dependence of strongly interacting NiO nanoparticles, we observed both a spontaneous exchange bias and vertical-loop shift [9]. The spontaneous exchange bias effect resulted from the setting up of unidirectional anisotropy across frustrated surface spins and the uncompensated antiferromagnetic core of NiO nanoparticles during the first field of hysteresis loop measurement, and it decreased with an increase of particle size. The observed vertical-loop shifts from bare NiO nanoparticles hint at the presence of strongly pinned spins at the interface of frustrated surface spins and the uncompensated antiferromagnetic core, which can further enhance the magnetic anisotropy.

The magnetic anisotropy plays an important role in shaping the magnetic properties of nanoscale material which can be altered by the random size distribution, strong interparticle interactions, and unidirectional anisotropy in the core/shell magnetic nanoparticles [16, 17]. The effect of enhanced magnetic anisotropy on the magnetic properties of nanoparticles can be studied effectively by measuring the thermoremanent spin dynamics [18], the memory effect [19], and the effect of aging [20] using field-cooling and zero-field-cooling processes. The field-cooling and zero-field-cooling memory effects have been used for the characterization of superparamagnetic [21] and spin-glassy [19] systems. The superparamagnetic system of nanoparticles shows weak field-cooling aging whereas the spin-glassy system shows aging with both field cooling and zero-field cooling which varies with the strength of the interparticle interaction. The memory effect has been observed and studied in ferromagnetic/antiferromagnetic core/shell nanoparticles [22], as well as ferromagnetic [23], ferrimagnetic [17], and antiferromagnetic [24] systems. Among these, antiferromagnetic materials at the nanoscale are of prime interest as showing drastic change in their magnetic properties varying from ferromagnetic, superparamagnetic, and paramagnetic to spin-glassy like. The spin-glassy-like behavior observed from NiO nanoparticles is co-related to strong interparticle interactions [24, 25]. However, in previous work [26], strongly interacting Fe_3_O_4_ nanoparticles which behave like spin glass and show both field-cooling and zero-field-cooling memory effects become non-interacting after reaching an interparticle spacing of 31.5 nm and exhibit field-cooling memory effect only. The above finding was also confirmed from our recent work on bare, weakly interacting 10-nm-size Fe_3_O_4_ nanoparticles which showed field-cooling memory effect only [17]. Therefore, whether the spin-glassy-like behavior of NiO nanoparticles is a collective phenomenon arising from the interparticle interaction or whether this is a true spin-glassy system still needs to be investigated. Note that the spin-glassy behavior can also arise because of frustrated spins at the surfaces of the particles [27, 28] or due to random freezing of the surface spin [29]. In this study, details of the synthesis, as well as the structural and magnetic properties of 14-nm NiO nanoparticles, are presented. The aim of this work is to investigate the effect of interparticle interactions and unidirectional anisotropy on the field-cooling and zero-field-cooling memory effect.

## Methods

The NiO nanoparticles used in this study are prepared using a sol–gel method, and the details of the synthesis process have been given in a previous report [10]. A homogeneous solution of 0.4 M nickel nitrate hexahydrate in ethanol is prepared at 50 °C by the application of continuous magnetic stirring for 2 h. Then, a uniform solution of 0.08 M oxalic acid dissolved in ethanol is added dropwise to the nitrate solution under continuous magnet stirring, resulting in the formation of a light cyan-colored gel. The gel is dried overnight in an ambient atmosphere. The dried powder was then ground to be used as a precursor in the preparation of different-sized nanoparticles. These were produced by annealing in a tube furnace at different temperatures ranging from 400 to 800 °C for a fixed duration of 1 h in air. Observations showed that the nanoparticles changed color from black to green with an increase of the annealing temperature. The nanoparticles were examined by field emission scanning electron microscopy (FE-SEM, JEOL JSM-6500F, JEOL, Japan) and transmission electron microscopy (TEM, JEM-3010, JEOL, Japan, working at 200 kV) [8] to determine the size distribution, morphology, and structure. Crystal structural analysis was carried out at the synchrotron radiation X-ray diffraction (SRXRD) facility at the National Synchrotron Radiation Research Center (NSRRC) in Hsinchu, Taiwan (beam line BL01C2), and XANES spectroscopy of the Ni *K*-edge was performed using the beam line BL01C1 [10]. The obtained XRD spectrum was analyzed by the Rietveld method [30] using a General Structure Analysis System (GSAS) software package [31].

## Results and Discussion

### Structural Analysis

**Fig. 1:**
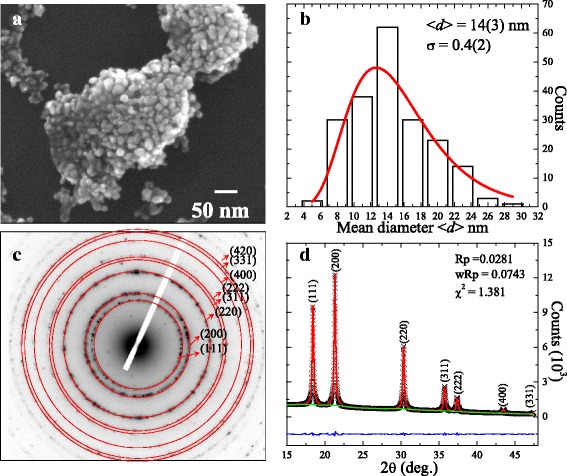
**a** SEM image of NiO nanoparticles showing the agglomeration behavior. **b** Histogram of the mean diameter distribution of nanoparticles obtained from the SEM images, where the *solid line* represents the fitting curve, assuming a log-normal distribution function. **c** SAED pattern and **d** Rietveld-refined (*red solid line*) synchrotron XRD pattern of NiO nanoparticles

The SEM image of the 400 °C annealed sample illustrated in Fig. 1a shows the formation of nanoparticles having a pseudo-spherical morphology and exhibiting an agglomeration behavior. Figure 1b depicts the histogram of the mean size distribution obtained after counting the diameters of around 200 nanoparticles from the SEM images. The observed asymmetric distribution of the nanoparticles can be fitted by assuming a log-normal distribution function, $f(d)=\frac{1}{{\left(2\pi \right)}^{1/2} d\sigma} \exp \left[-\frac{{\left( \ln d- \ln \left\langle d\right\rangle \right)}^2}{2{\sigma}^2}\right]$, where <*d*> is the mean diameter and *σ* is the standard deviation of the fitted function. The fitted value of the mean diameter <*d*> and standard deviation *σ* of the nanoparticles is 14 ± 3 nm and 0.4 ± 0.2, respectively. The observed wide distribution and agglomeration behavior of the nanoparticles can significantly affect the magnetization properties. The SAED pattern of the nanoparticles, as shown in Fig. 1c, exhibits electron diffraction spots in a ring pattern, indicating the formation of NiO nanoparticles which are polycrystalline. The ring pattern of the electron diffraction spots is attributed to the indexed (111), (200), (220), (311), (222), (400), (331), and (420) peaks based on face-centered-cubic (fcc) $F m\overline{3} m$ NiO. No additional diffraction spots other than the NiO phase are observed [11]. The XRD spectrum of the nanoparticles shown in Fig. 1d confirms the formation of pure NiO without any trace of impurity. A significant broadening of the nuclear peak shown in the XRD spectrum is an indication of the nanometric nature of the sample. The observed broadening of the nuclear peak can be described using the Gaussian distribution function. The value of the grain size is calculated using the Scherer formula from the full width at the half maximum (FWHM) 0.260 ± 0.004° of the most intense peak (200) to be 16 ± 2 nm, which is in good agreement with the mean diameter estimated from SEM. To estimate the effect of the finite size on the lattice parameter, we also carried out Rietveld refinement [30] using the GSAS software package [31]. The solid red line is the fitted curve to the diffraction pattern represented by crosses in Fig. 1d. The difference between the observed and fitted patterns is indicated by the blue line at the bottom of the figure while the green line shows the fit to the background. The fitted values of the lattice parameter *a* = *b* = *c* = 4.1900 ± 0.0004 Å confirm the fcc NiO phase. The observed lattice expansion of the nanoparticles, compared to the bulk value of 4.1710 Å, is ascribed to finite size effect and the nickel vacancies (detailed description of size dependence of EXAFS can be found in Additional file 1: Figures S1) [8, 10, 32].

### Static-Magnetization Measurements

**Fig. 2:**
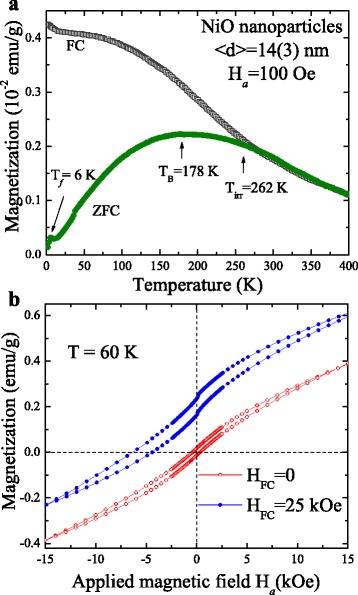
**a** ZFC–FC magnetization curve of 14 nm NiO measured in a field of 100 Oe. **b** ZFC–FC hysteresis loop of nanoparticles measured at 60 K

**Fig. 3:**
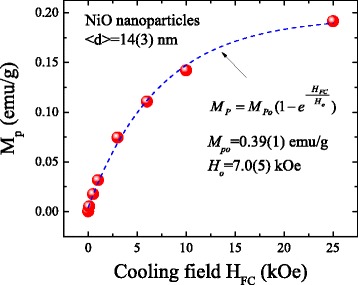
Plot of calculated values of effective magnetic moment versus cooling field. The *blue dashed curve* indicates the fit of the data to the theoretical curve for an exponential decay function

Prior to magnetic characterization, the powdered sample was then packed into a thin magnetic cylindrical holder. The magnetic properties of the nanoparticles were measured with a superconducting quantum interference device (Quantum Design, MPMS-VSM) magnetometer. Figure 2a presents the variation of the magnetization *M*(*T*) with temperature, measured under an applied magnetic field of 100 Oe using the zero-field-cooled (ZFC) and field-cooled (FC) processes. The pronounced broad peak temperature that appears on the ZFC magnetization curve near 178 K is associated with the blocking temperature of superparamagnetic NiO nanoparticles. An irreversible temperature *T*_irr_ = 262 K and a freezing temperature *T*_f_ = 6 K were observed from the *M*(*T*) curves [9]. The *T*_f_ is associated with collective freezing of uncompensated surface spins of NiO [8]. The spins at the surface of the 14-nm NiO nanoparticles are highly disordered [10]. Assuming that the above system displays superparamagnetic properties, we therefore used the Néel-Brown model [33] *T*_B_ = *KV*/22*k*_B_ (where *V* is the volume of the particles and *k*_B_ is the Boltzmann constant) to estimate the magnetic anisotropy energy density *K*. The calculated value of *K* for 14 nm NiO is 225,852 erg/cm [3], which is 3.1 times that of the value for a non-interacting nanoparticle system [8]. The observed enhancement in the value of *K* could be due to the broad size distribution of the nanoparticles, the strong interparticle interactions, and the interfacial strongly pinned spins. The ZFC magnetization hysteresis *M*(*H*_*a*_), measured at 60 K, as shown in Fig. 2b, reveals an asymmetric and horizontal- and vertical-loop shift. The obtained values of the remanence ${M}_{\mathrm{r}}=\left({M}_{\mathrm{r}}^{+}-{M}_{\mathrm{r}}^{-}\right)/2$, coercivity ${H}_{\mathrm{C}}=\left({H}_{\mathrm{C}}^{+}-{H}_{\mathrm{C}}^{-}\right)/2$, spontaneous exchange bias (SEB) field ${H}_{\mathrm{SEB}}=\left({H}_{\mathrm{C}}^{+}+{H}_{\mathrm{C}}^{-}\right)/2$, and vertical-loop shift ${M}_{\mathrm{vls}}=\left({M}_{\mathrm{r}}^{+}+{M}_{\mathrm{r}}^{-}\right)/2$ are 0.0153 emu/g, 373 Oe, −60 Oe, and 0.0023 emu/g, respectively. Here, ${H}_{\mathrm{C}}^{+}\ \left({M}_{\mathrm{r}}^{-}\right)$ and ${H}_{\mathrm{C}}^{-}\ \left({M}_{\mathrm{r}}^{+}\right)$ indicate the coercive fields (remanence) for the ascending and descending curves, respectively. The observed non-zero values of *M*_r_ and *H*_C_ confirm the FM behavior of the 14-nm NiO nanoparticles originating from the nickel vacancies and finite size effect. The observed SEB effect arises from inter-coupling between the short-range ordered clusters of frustrated surface spins and uncompensated antiferromagnetic cores at the interface during the first field of hysteresis loop measurement. Such inter-coupling sets up unidirectional anisotropy at the interface; for a detailed discussion, please see our previous work [9]. The observed shift in the vertical loop in the zero field could be due to presence of strongly pinned spins at the interface such that the magnetic field cannot reverse. The irreversible behavior of the strongly pinned spins is confirmed by the highly asymmetric behavior of the field-cooled *M*(*H*_*a*_) loop, which shifts significantly upward, in the direction of the cooling field by +25 kOe, as shown in Fig. 2b. The magnitude of the magnetization in the positive field direction (+15 kOe) is larger than that in the negative field direction (−15 kOe), which can be understood by considering that part of the moments of nanoparticles possess strongly pinned magnetization (*M*_p_) in the FC direction, but the field cannot be reversed. The net moment of these interfacial pinned spins can be quantified as follows: *M*_p_ = 1/2Δ*M*, where Δ*M* = [*M*(+15 kOe) − *M*(−15 kOe)]. The observed non-zero value of *M*_p_ = 0.0004 emu/g in the zero cooling field can further mediate the unidirectional anisotropy during the first field of hysteresis loop measurement. In Fig. 3, the calculated values of *M*_p_ versus the applied cooling field *H*_FC_ are plotted, revealing an increase with the increase in the cooling field *H*_FC_. The *blue dashed curve* indicates the fit of the data to the theoretical curve for an exponential decay function, namely ${M}_{\mathrm{P}}={M}_{\mathrm{P}\mathrm{o}}\left(1-{e}^{-\frac{H_{\mathrm{FC}}}{H_{\mathrm{o}}}}\right)$, where *M*_Po_ = 0.39 (1) emu/g and *H*_o_ = 7.0(5) kOe represent the initial constant and the fitted parameters, respectively. This result shows that the strength of UA is dependent on the net moment of the strongly pinned interfacial spins and the concentration of uncompensated spins and therefore should decrease with an increase in the nanoparticle size. Therefore, the above observation indicates that the spontaneous exchange bias effect is mediated by the strongly pinned interfacial spins [9].

### Magnetic Relaxation

**Fig. 4:**
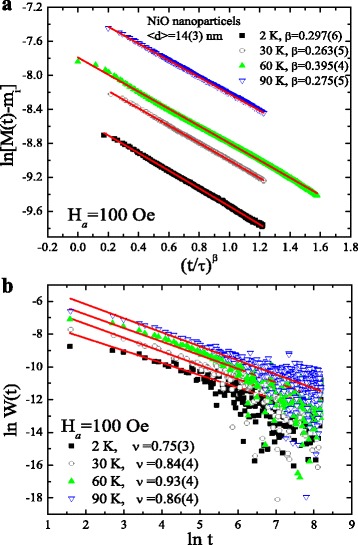
**a** Stretched-exponential and **b** power-law fit to the magnetization relaxation curve measured for 1 h from 14 nm NiO measured at 2, 30, 60, and 90 K in a 100-Oe field. The fitting parameters are depicted in the figure

The time dependency of the magnetization relaxation *M*(*t*) was measured at 2, 30 60, and 90 K (in the blocked state) for a time period of 3600 s. The *M*(*t*) measurement was carried out by first cooling the system to the desired temperature under a small magnetic field of 100 Oe. Once the temperature stabilized, the magnetic field was turned to oscillator mode at a rate of 10 Oe/s, and the subsequent relaxation of the magnetic moment with respect to time was recorded. Figure 4a depicts the plot of ln[*M*(*t*) − *m*_i_] verses *t* ^*β*^. The fitted value of *β* lies between 0.3 and 0.4 with the highest value at 60 K. The solid lines in Fig. 4a represent the fit to the relaxation dynamics using the stretched exponential function defined as follows: *M*(*t*) = *m*_i_ − *m*_g_ exp(−(*t*/*τ*)^*β*^), where *m*_i_ is an intrinsic magnetic component, *m*_g_ is the glassy component, *τ* is the characteristic relaxation time, and *β* is a stretching parameter which is a function of the measuring temperature [34]. In the above expression, the value of *β* defines the activation against the single (*β* = 1) or multiple anisotropy barriers (0 < *β* < 1). The fitted value of *β* points toward activation against multiple anisotropy energy barriers. The possible causes for such a low value of *β* could be the size distribution, strong interparticle interactions, shape anisotropy, spin-glass phase, and the interfacial pinned-spin-mediated UA. For a better understanding, the above *M*(*t*) curves were further examined using the theoretical model proposed by Ulrich et al. [18]. The modeling explicitly showed that the decay of *M*(*t*) followed a power law after the lapse of crossover time *t*_c_: *W*(*t*) = *t* ^−*ν*^, where *W*(*t*) = −(d/dt)ln*M*(*t*). The value of *ν* is a function of the measuring temperature. A plot of *W*(*t*) verses ln*t* is shown in Fig. 4b where the straight line displays the power-law fit after a crossover time *t*_c_ = 100 s. The fitted value of *ν* is higher than two thirds, indicating a dense system of nanoparticles, which is in agreement with the agglomeration behavior observed in the SEM images. The above findings suggest the presence of strong interparticle interaction. The value of *ν* increases from 0.75 to 0.93 with an increase in the temperature from 2 to 60 K and then decreases to 0.86 at 90 K.

### Memory Effect

**Fig. 5:**
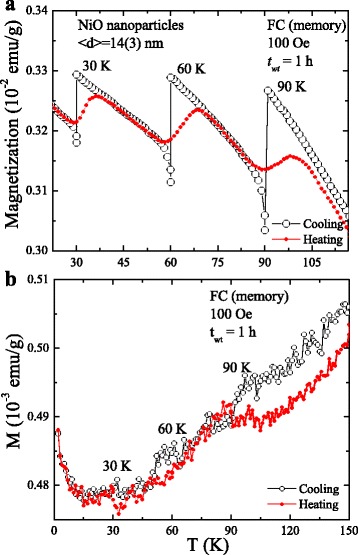
**a**, **b** FC memory effect measured from 14- and 54-nm NiO nanoparticles in the 100-Oe field with a halt of 1 h at 30, 60, and 90 K

**Fig. 6:**
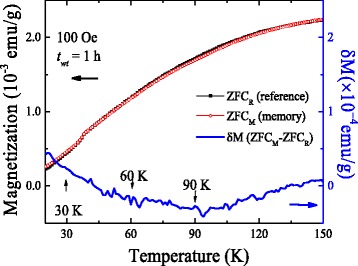
ZFC memory effect and thermal variation of the difference magnetization δ*M* measured from 14-nm NiO nanoparticles in the 100-Oe field with a halt of 1 h at 30, 60, and 90 K

We used the same protocol as suggested by Sun et al. [35], attributing memory effect to an interacting magnetic nanoparticle system. The nanoparticles were first cooled down in a magnetic field of 100 Oe with sporadic stops with a zero field at a few selected temperatures, 250, 200, 150, 100, 70, 40, and 10 K, for a time of *W*_*t*_ = 5 h before cooling was resumed. The magnetization was recorded during this “cooling” process. After reaching the desired low temperature of 2 K, the sample was warmed and magnetization recorded, called the “heating” process. The memory effect on the thermal variation of the magnetization in the strongly interacting 14-nm and non-interacting AFM-like 54-nm NiO nanoparticles is investigated using the FC process in *H*_*a*_ = 100 Oe, as shown in Fig. 5a, b. Initially, the sample was cooled from 150 to 2 K at a rate of 2 K/min while the magnetization was measured. Cooling was temporarily stopped at 90, 60, 30, and 2 K for a period of 1 h each time. During each stop, the applied magnetic field was turned off in oscillatory mode at a rate of 10 Oe/s to allow the magnetic moments to relax to a zero magnetic field. After completion of each stop, the magnetic field was re-applied and measurement was subsequently resumed until the temperature reached 2 K. After this, the sample was then reheated under 100 Oe without any halt. The produced magnetization curve is the so-called magnetic memory curve *M*_mem_ (indicated by the red solid circles), as shown in the Fig. 5a. One can observe three discernible steps around the halting temperatures in the *M*_mem_ curve, although measurement was carried out without any halt; see Fig. 5a. This observed step-like behavior is a sign of the memory effect in which the spin configuration imprinted at each temperature halt is retrieved as it appears on the curve during heating the sample. The observed FC memory effect decreases with increasing particle sizes and disappears when the nanoparticles exceed ~30 nm NiO (see Additional file 1: Figures S2(a)–(c)). Figure 5b shows the absence of the memory effect in the 54-nm NiO nanoparticles. The unique part of the discernible steps observed in Fig. 5b is the increase in the magnetization of the FC curve with the decrease in temperature below 30 K. This behavior is commonly assigned to a non-interacting superparamagnetic system [21]. In general, an interacting spin-glassy system shows a decrease in the magnetization behavior with a decrease in the measuring temperature [19]. The above discrepancy can be resolved by measuring the ZFC memory effect. During the ZFC process, the sample is initially cooled down from 150 to 15 K at a rate of 2 K/min in a zero field, halting at 90, 60, and 30 K for a time period of 1 h each. During warming, the magnetization is recorded in a 100-Oe field. From previous reports, it is well understood and established that the ZFC memory effect can only be observed either with interacting nanoparticles or a spin-glassy-like system, but not from non-interacting superparamagnetic systems. However, we note that the memory effect was absent in the thermal variation of the ZFC magnetization curve recorded when the sample was cooled down to 2 K from 150 K without the application of a magnetic field. The thermal variation of the difference in the magnetization δ*M* (δ*M* = ZFC_M_ − ZFC_R_) between the ZFC_M_ (memory) and ZFC_R_ (reference) is plotted in Fig. 6. During the ZFC process, the sample temperature stabilized at 100 K, as shown in the ZFC magnetization plot, which is also clearly highlighted in the difference plot, i.e., no memory is imprinted by aging in the zero field for the 1-h time period. The difference in magnetization data (blue solid line) increase slowly at temperatures lower than 90 K, indicating that the magnetic moment configuration spontaneously rearranges toward equilibrium as their correlation length increases. This implies that the correlation between the nanosized Ni moments develops in a similar way as the correlation between the spins in spin glasses, as the temperature is below 90 K. Therefore, when interpreting the dynamic behavior of these interacting NiO nanoparticles, the effects of the spin-glass-like correlations may have to be taken into account which seem to favor the spin-glass hypothesis explaining the observed collectivity and glassiness. The absence of memory in the ZFC mode indicates that either the system is superparamagnetic or the interparticle interaction is too weak to retain the ZFC memory effect. In a spin-glassy system, the length of the spin–spin correlation grows during the stop, even in a zero field, and a memory dip typically shows up upon reheating. This is not possible in a non/weak-interacting nanoparticle system which does not show the memory dip in the ZFC mode. The above finding indicates that the FC memory effect in strongly interacting 14-nm NiO nanoparticles could be induced either by the size distribution (which resulted in the broad distribution of blocking temperatures) [36] or the strongly pinned interfacial spin-mediated UA. However, the observed decreasing behavior of both the FC memory effect and the SEB field with the increase in the NiO nanoparticle size strongly indicates that the memory effect in NiO nanoparticles is mediated by strongly pinned interfacial spins.

## Conclusions

The detailed magnetic and structural properties of 14-nm NiO nanoparticles are systematically studied. The zero-field-cooled hysteresis loop reveals their ferromagnetic behavior and effective spontaneous exchange bias field of 60 Oe, due to the setting up of unidirectional anisotropy during the first field of hysteresis loop measurement. The analysis of a series of field-cooled *M*(*H*) reveals that the observed spontaneous exchange bias and unidirectional anisotropy is mediated by strongly pinned spins at the interface of frustrated surface spins and uncompensated antiferromagnetic cores. The magnetization relaxation measured at different temperature shows the presence of multimagnetic anisotropy in the nanoparticles, which could possibly originate from strong interparticle interactions, broad size distributions, spin-glass behavior, and unidirectional anisotropy. However, observed memory effect only occurs when the field-cooling process has the characteristics of an (i) increase of magnetization with decrease of temperature and (ii) the fade out behavior with an increase of particle size rules out the possibility of the effect of strong interparticle interactions, broad size distributions, and spin-glass behavior. We conclude that the observed field-cooling memory effect from bare, small size NiO nanoparticles arises because of the unidirectional anisotropy which is mediated by the interfacial strongly pinned spins.

## Additional file

Additional file 1: Figure S1. Ni *K*-edge EXAFS spectra *χ*(*k*)*k* ^2^ and their Fourier transforms (FTs) for a series mean size of the NiO nanoparticles from 14 to 31 nm, respectively. Figure S2 (a)–(c). The FC memory effect measured from 19, 29, and 31 nm NiO in the 100-Oe field with a halt of 1 h at 30, 60, and 90 K, respectively, showing the fading of the memory effect with the increase of particle size. (DOCX 298 kb)

## Abbreviations

AFM: Antiferromagnetism
FC: Field-cooled
FE-SEM: Field emission scanning electron microscopy
FM: Ferromagnetism
SEB: Spontaneous exchange bias
TEM: Transmission electron microscopy
XRD: X-ray diffraction
ZFC: Zero-field-cooled
