# Erratum to: Strong Pinned-Spin-Mediated Memory Effect in NiO Nanoparticles

**DOI:** 10.1186/s11671-017-2090-0

**Published:** 2017-05-05

**Authors:** Ashish Chhaganlal Gandhi, Ting Shan Chan, Jayashree Pant, Sheng Yun Wu

**Affiliations:** 1 Department of Physics, National Dong Hwa University, Hualien, 97401 Taiwan; 2 Center for Condensed Matter Sciences, National Taiwan University, Taipei, Taiwan; 3 National Synchrotron Radiation Research Center, Hsinchu, 30076 Taiwan; 4 Department of Physics, Abasaheb Garware College, Savitribai Phule Pune University, Pune, India

## Erratum

In the original publication [1] was an error in the authors contribution. The correct version can be found here:

“SYW and ACG wrote, conceived, and designed the experiments. ACG and JP grew the samples and analyzed the data. TSC and JP contributed the experimental facilities and valuable discussions. All authors discussed the results, contributed to the manuscript text, commented on the manuscript, and approved its final version.”
