# Supplementary material for: Strong Pinned-Spin-Mediated Memory Effect in NiO Nanoparticles
Source: Nanoscale Res Lett. 2017 Mar 21;12:207. doi: 10.1186/s11671-017-1988-x (PMC5359196; doi:10.1186/s11671-017-1988-x)
Supplement: Additional file 1: Figure S1. — Ni K-edge EXAFS spectra χ(k)k 2 and their Fourier transforms (FTs) for a series mean size of the NiO nanoparticles from 14 to 31 nm, respectively. Figure S2 (a)–(c). The FC memory effect measured from 19, 29, and 31 nm NiO in the 100-Oe field with a halt of 1 h at 30, 60, and 90 K, respectively, showing the fading of the memory effect with the increase of particle size. (DOCX 298 kb) [file 11671_2017_1988_MOESM1_ESM.docx]

**Supplementary Information**

**Strong pinned spins mediated memory effect in NiO nanoparticles**

Ashish Chhaganlal Gandhi,^ab^ Ting Shan Chan,^c^ Jayashree Pant,^d^ and Sheng Yun Wu^*a^

^a^Department of Physics, National Dong Hwa University, Hualien, Taiwan

^b^Center for Condensed Matter Sciences, National Taiwan University, Taipei, Taiwan

^c^National Synchrotron Radiation Research Center, Hsinchu 30076, Taiwan

^d^Department of Physics, Abasaheb Garware College, Savitribai Phule Pune University, Pune, India

**Figure S1** Ni *K*-edge EXAFS spectra χ(k)k^2^ and their Fourier transforms (FTs) for a series mean size of the NiO nanoparticles from 14 nm to 31 nm, respectively.

**
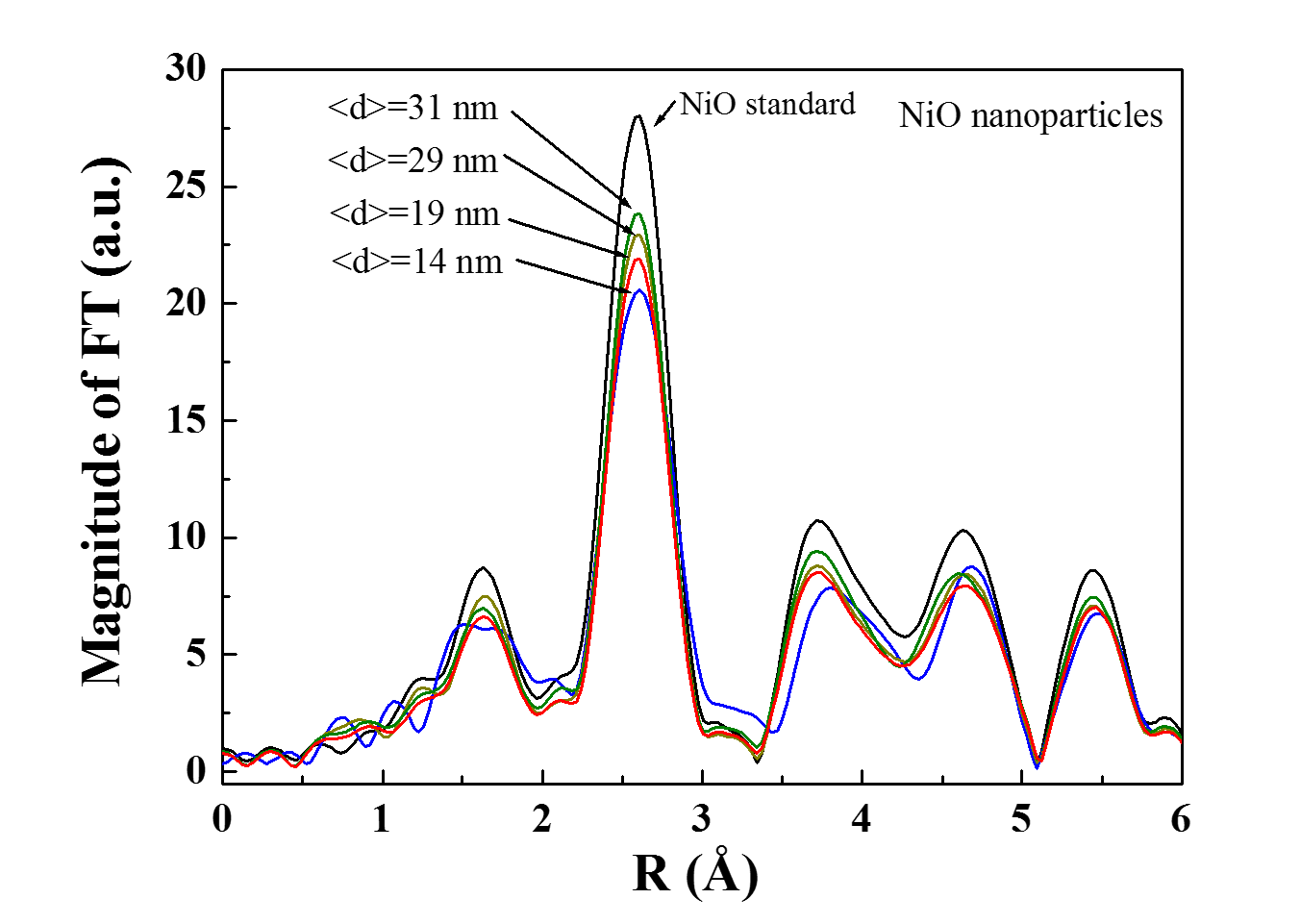
**

XANES spectroscopy of the Ni *K*-edge was performed using the beam-line 01C1 of the National Synchrotron Radiation Research Center (NSRRC) in Hsinchu, Taiwan. The generation of a synchrotron radiation source originates from a deflection of the motion of charged particles by a magnetic field, which releases electromagnetic radiation in the shape of a narrow beam along a tangential direction. This electromagnetic radiation is called “synchrotron radiation”. As the synchrotron radiation source transports to the BL01C1 experiment station, the synchrotron light source passes first through the fixed-exit Si(111) double crystal monochromator (DCM) which provides an energy range from 6 keV to 33 keV. The energy resolution reaches to 2.3 × 10^-4^ @20keV for the light source. Thus, the BL01C1 light source is useful for analyzing and distinguishing the structural types of Ni and NiO covering the energy range from 8000 to 8400 eV for the Ni *K*-edge. EXAFS (Extended X-ray Absorption Fine-Structure) indicates that x-rays are absorbed by a core electron in the atom. The energies are above the core level binding energies, which can provide information about the local atomic structure around the absorbing atom. For the influence of extra oxygen, EXAFS analysis can support an accurate examination of the core-shell structure in the vacancies concentration. Figure S2 show Ni *K*-edge EXAFS spectra χ(k)k^2^ and their Fourier transforms (FTs) for a series mean size of the NiO core-shell nanoparticles from 14 nm to 31 nm, respectively. The Ni *K*-edge EXAFS spectra were calculated for each atomic configuration using Athena and Artemis within the framework of FEFF’s multiple-scattering path expansion. The height for main Ni peak at R=2.607 $Å$ rapidly increases from 20.4 to 23.8 with increasing the mean size. It can be seen that our nanocrystalline samples give strong evidence of the of nickel vacancies and size distribution, which corresponds and agrees to previous reports of nickel vacancies in the nanoscale [8].

**
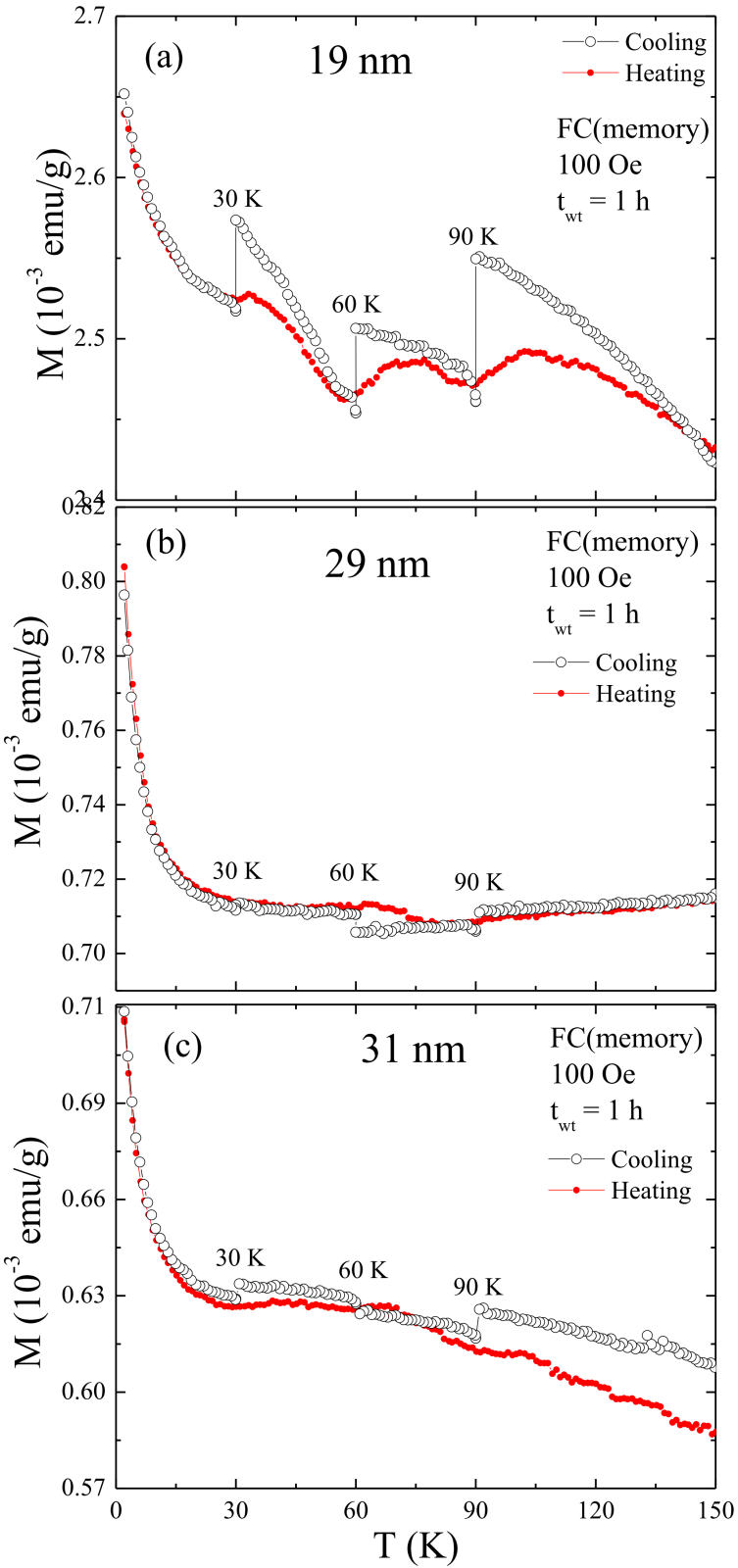
Figure S2 (a)-(c)** The FC memory effect measured from 19, 29 and 31 nm NiO in the 100 Oe field with a halt of 1 h at 30, 60 and 90 K, respectively, showing the fading of the memory effect with the increase of particle size.
